# Upgrade of *D*+ software for hierarchical modeling of X-ray scattering data from complex structures in solution, fibers and single orientations

**DOI:** 10.1107/S1600576723005319

**Published:** 2023-07-28

**Authors:** Eytan Balken, Itai Ben-Nun, Amos Fellig, Daniel Khaykelson, Uri Raviv

**Affiliations:** a Institute of Chemistry, The Hebrew University of Jerusalem, Edmond J. Safra Campus, Givat Ram 9190401, Jerusalem, Israel; b Department of Molecular Chemistry and Materials Science, Weizmann Institute of Science, Rehovot 76100, Israel; c Center for Nanoscience and Nanotechnology, The Hebrew University of Jerusalem, Edmond J. Safra Campus, Givat Ram 9190401, Jerusalem, Israel; Argonne National Laboratory, USA

**Keywords:** X-ray scattering, fiber diffraction, hierarchical modeling, *D*+ software, structure factors, polydispersity, orientations

## Abstract

This article presents an upgrade of the *D*+ software [Ginsburg *et al.* (2019 ▸). *J. Appl. Cryst.* **52**, 219–242], expanding its hierarchical solution X-ray scattering modeling capabilities for fiber diffraction and single crystallographic orientations. This upgrade was carried out using the reciprocal grid algorithm [Ginsburg *et al.* (2016 ▸). *J. Chem. Inf. Model.* **56**, 1518–1527], providing *D*+ its computational strength. Furthermore, the extensive modifications made to the Python API of *D*+ are described, broadening the X-ray analysis performed with *D*+ to account for the effects of the instrument-resolution function and polydispersity. In addition, structure-factor and radial-distribution-function modules were added, taking into account the effects of thermal fluctuations and intermolecular interactions. Finally, numerical examples demonstrate the usage and potential of the added features.

## Abstract 2

An upgrade to the *D*+ software is presented, simulating the 2D scattering pattern from structures with a single orientation or fibers. An upgraded independent *D*+ Python API is described, including structure factor, polydispersity and several other features.

## 1. Introduction

X-ray scattering is an important tool for determining mol­ecular structures and intermolecular interactions. In an X-ray scattering experiment, the scattering intensity, *I*, is measured as a function of the scattering vector, **q**, given by **q** = (*q*_*x*_, *q*_*y*_, *q*_*z*_) = (*q*, θ_*q*_, ϕ_*q*_), in Cartesian and spherical coordinates, respectively. The scattering intensity is the square of the scattering amplitude, *F*, given by 

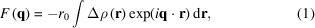

where **r** is the position vector in real space; Δρ(**r**) is the electron-density contrast of the scattering particles, with respect to the medium, as a function of **r**; and *r*_0_ = 2.82 × 10^−5^ Å is the Thomson scattering length (Als-Nielsen & McMorrow, 2011 ▸). In solution, an orientation average over the reciprocal-space solid angle, Ω_*q*_,

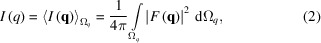

should be computed. The term 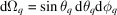 , where θ_*q*_ and ϕ_*q*_ are the reciprocal (**q**)-space polar and azimuthal angles, respectively.

Much effort has been devoted to scattering data analysis and modeling. With the evolution in the strength of computers came the evolution of X-ray technology, and the complexity of the experiments followed suit. As previously shown (Ginsburg *et al.*, 2019 ▸, 2016 ▸), the analysis program *D*+ (https://scholars.huji.ac.il/uriraviv/book/d-0), developed in our laboratory, can accurately and rapidly compute the expected solution small/wide-angle X-ray scattering intensity from highly complex and large structural models.

In *D*+, structures can be defined in hierarchical data-structure trees, using geometric or atomic model subunits forming the tree’s leaves. Repeating subunits are then docked into their assembly symmetries (the tree’s nodes), containing the locations and orientations of repeating subunits. The scattering amplitude of the entire structure, made of *J* unique subunits, is

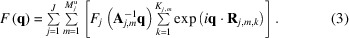




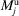
 is the number of unique orientations of an object of type *j*, given by the Tait–Bryan rotation matrices **A**_*j*,*m*_. Furthermore, *K*_*j*,*m*_ is the number of real-space translations, **R**_*j*,*m*,*k*_, of object *j* with orientation **A**_*j*,*m*_. In *D*+, *F*(**q**) can be calculated by computing the scattering amplitudes of the subunits on 3D reciprocal-space grids. When moving up in the hierarchy, the reciprocal grids of larger structures are computed by interpolating precomputed lower-level reciprocal grids. The final scattering amplitude is obtained by repeating this process for all the leaves and nodes of the tree data structure.

In the direct method, no grids are computed, and the scattering amplitude is directly computed by summing all the subunit contributions in the complex structure [using equation (3)]. For very large structures, a hybrid method can be used. In this method, only grids of smaller subunits are summed and used as subunits (*i.e.* leaves in the tree data structure) in a direct computation of the scattering amplitude [equation (3)] for the nodes in the hierarchy for which grids are not computed (Ginsburg *et al.*, 2019 ▸). The orientation average [equation (2)] is then numerically calculated by selecting many random angles in reciprocal space until the scattering intensity converges.

The *D*+ program has been used to analyze steady-state and time-resolved solution X-ray scattering data from various complicated structures at high resolution (Asor *et al.*, 2017 ▸, 2020*a* ▸; Shaltiel *et al.*, 2019 ▸; Dharan *et al.*, 2021 ▸; Ginsburg *et al.*, 2017 ▸). In addition, to analyze an ensemble of dynamic structures and to take into account their stability, *D*+ has been integrated with Monte Carlo simulations (Louzon *et al.*, 2017 ▸; Asor *et al.*, 2019 ▸), the thermodynamic theory of macromolecular self-assembly (Asor *et al.*, 2019 ▸; Shemesh *et al.*, 2021 ▸), rate equations (Shemesh *et al.*, 2022 ▸) and maximum information entropy optimization (Asor *et al.*, 2020*b* ▸).

Since the release of version 4.1, in 2019, several changes have been made to *D*+. We have upgraded the versions of CUDA (11.7) (https://www.nvidia.com/en-gb/geforce/technologies/cuda/), Python (3.8, 3.9, 3.10, 3.11), *Visual Studio* (2022) and *Ceres Solver* (2.0.0) (http://ceres-solver.org/), improved the error messages, created a more intuitive user interface, added GitHub automation to create the installer and the Python wheels, added tests, introduced JSON file format (https://www.json.org/), fixed several bugs, considerably improved the internal workflow of the program, and updated the Python API of *D*+ (https://scholars.huji.ac.il/uriraviv/book/python-api), making it independent of the installation of *D*+. We have also implemented the option to account for instrument resolution, similarly to how it is done in the *X*+ software (Ben-Nun *et al.*, 2010 ▸). Other changes, however, have taken *D*+ to the next level. We have built a Python API module that receives a list of *N* repeating subunit positions, **r**_*i*_ (*i.e.* a structural model), and computes the structure factor, 

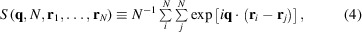

its orientation average,

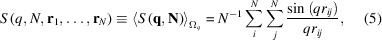

where *r*_*ij*_ ≡ |**r**_*i*_ − **r**_*j*_|, and the radial distribution function, 

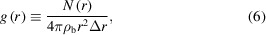

where ρ_b_ is the average bulk subunit number density. *N*(*r*) is the number of repeating subunits in a shell of radius *r* and thickness Δ*r*, from a random subunit *r*_*i*_, averaged over all the subunits in the list, applying periodic boundary conditions. The module can also compute *g*(*r*) from *S*(*q*) and *S*(*q*) from *g*(*r*). Our most important upgrade is the option to compute the 2D scattering intensity pattern from a structure in a specific orientation, as typically measured by area detectors. In addition, we have created a module for computing the 2D fiber diffraction pattern from a fiber containing subunits with a uniform azimuthal angle distribution within a fiber aligned in a specific direction. This module performs orientation averaging over the reciprocal-space azimuthal angles, ϕ_*q*_. Finally, in the Python API of *D*+, we have implemented the possibility of simulating the effects of thermal fluctuation and polydispersity in each parameter. All of these changes will be discussed and demonstrated in the following sections. The examples we shall present will provide insights into the functions and the correct usage of the Python API of *D*+.

## 2. Materials and methods

### 2.1. The Python API of *D*+

The Python API of *D*+ is a fully independent pythonic version of the *D*+ software, meaning that one can install the API without having to install the entire *D*+ software with its graphical user interface (GUI). Using the Python API of *D*+, the user can exploit and combine all the main functions of *D*+ as needed, and integrate them into any other code. The opposite is also true. One can integrate all the Python modules (like *NumPy* or *SciPy*; https://numpy.org/; https://scipy.org/) to build and simulate models, requiring advanced and sophisticated analyses.

The *D*+ program is broken down into different modules, providing all the computational functions of *D*+. Thus, we have the CalculationInput module, with which one can build the ‘state file’, containing all the information required for computing a model. In addition, the module DataModels(.models) covers all the structural models implemented in *D*+. After creating a model (and saving it in a state file), one calculates the scattering intensity of the model using the CalculationRunner module. In the end, if the model was run using a grid, one might want to use the Amplitude module for other purposes, like obtaining its 2D scattering pattern.

As the API has many functions, a document explaining all the inner workings of the Python API of *D*+ has been provided in our GitHub repository (API README; https://github.com/uri-raviv-lab/dplus-dev/blob/development/PythonInterface/README.md). Our code is open source for academic purposes, and fixes for encountered bugs or adding new features or functions are wholeheartedly accepted, as are any questions or problems encountered during installation or usage (*D*+ Issues, https://github.com/uri-raviv-lab/dplus-dev/issues; *D*+ GitHub, https://github.com/uri-raviv-lab/dplus-dev).

### 2.2. Resolution function

When performing an X-ray experiment, the scattering pattern might be different from what is computed from a model. One of the possible reasons is the finite resolution of the setup. We can take this effect into account, as done in the *X*+ program (Ben-Nun *et al.*, 2010 ▸), by convolving the modeled scattering intensity with a Gaussian resolution function, with a standard deviation given by σ. This function smears the model, meaning that some sharp peaks or minima will be less prominent or will merge with others (Pedersen *et al.*, 1990 ▸; Pauw, 2013 ▸). To simulate this effect, *D*+ has, next to the ‘Generate’ button, an input area for the standard deviation, σ, of a Gaussian instrument resolution function, offering users the choice of whether to take it into account or not. Similarly, a Gaussian resolution function with a specific σ can be added when building a state in the Python API of *D*+, using apply_resolution.

### 2.3. Polydispersity

In experiments, the measured particles often have a distribution of sizes rather than a single size. This effect can be modeled by adding a standard deviation to the geometric model parameters in the Python API of *D*+. In turn, as in *X*+ (Ben-Nun *et al.*, 2010 ▸), the model will be recalculated 14 times with a change of the size parameter, using the inputted standard deviation inside a Gaussian weighting distribution around the parameter’s center value (*i.e.* 15 models will be calculated in total). This function only works with geometric models, not atomic ones. The polydispersity of atomic models might be computed by averaging the solution scattering intensity of different conformations generated, for example, by a Monte Carlo simulation (Louzon *et al.*, 2017 ▸).

Using the Python API of *D*+, any other polydispersity weighting function (Zimm, 1948 ▸; Kotlarchyk & Chen, 1983 ▸; Breßler *et al.*, 2015 ▸) can be implemented for geometric or atomic models.

### 2.4. *g*(*r*) and *S*(*q*) modules

The following subsections are based on the functions inside the *g*(*r*) module, whose functions work, where relevant, with *NumPy* arrays (Harris *et al.*, 2020 ▸). The structure factor and the radial distribution function which are based on a structural model [equations (4 [Disp-formula fd5])–(6)] have been parallelized on CPUs using the *DaCe* algorithm (Ben-Nun *et al.*, 2019 ▸). The parallel computation allowed us to get results in a reasonable time, even for large models.

#### 2.4.1. Supporting functions

In this module, three important supporting functions were built:

(1) build_crystal is a function similar to the space-filling symmetry, which builds a docking list (‘dol’) file from either lattice vectors [**a**, **b**, **c**] or lattice constants [*a*, *b*, *c*, α, β, γ], and the number of repetitions in each direction. By default, the crystal is moved to its geometric center.

(2) thermalize adds uniformly distributed random fluctuations to the lattice points according to Δ**r** = 2**u** *v* − **u**, where *v* ∈ [0, 1] (randomly selected) and **u** is the maximal fluctuation in each coordinate. thermalize takes a list of points and adds normally distributed random fluctuations as defined by a user-inputted standard deviation. Unlike build_crystal, thermalize does not build a crystalline structure and then fluctuate the points, but rather takes an already built set of coordinates (that do not have to be crystalline) and adds fluctuations. In this way, it is possible to build different states that start from the same coordinates and differ only by a user-defined random factor.

(3) MC_Sim function is a Monte Carlo simulator, accounting for thermal fluctuations of a set of points (*i.e.* a dol file) by working against either harmonic or Lennard-Jones potentials (Jones, 1924*a* ▸,*b* ▸), *V*(*r*), between nearest neighbors, nn. The pairwise energy cost, 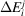 , of a random displacement 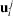 is

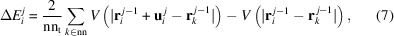

where nn_t_ is the total number of nearest neighbors. The factor of two originates from the fact that each interaction is shared between the interacting pair. The probability of obtaining a random displacement 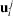 is

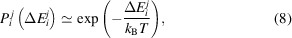

where *k*_B_ is the Boltzmann constant and *T* is the absolute temperature. Monte Carlo simulations estimate the effect of thermal fluctuations. At iteration *j*, the probability, 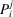 , of a random displacement, 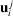 , at a random lattice point 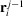 is compared against a random number between 0 and 1. The displacement is accepted if the random number is smaller than 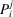 . This process is repeated until the maximum number of iterations (provided by the user) is attained while maintaining periodic boundary conditions. At the end of the process, a new dol file is saved with the new coordinates.

#### 2.4.2. Structure factor

The structure factor is an important property of crystals or oriented samples [*S*(**q**)] and solutions [*S*(*q*)]. It is needed for analyzing X-ray scattering data, as it sheds light on the arrangement of subunits in a sample (Yarnell *et al.*, 1973 ▸). Therefore, four functions were built to compute the structure-factor contribution, assuming monodispersed particles:

(i) Amp_of_SF receives a model as a dol file containing a list of subunit positions (a docking list generated, for example, using one of the supporting functions). The function computes the theoretical structure factor associated with the model in a single orientation [equation (4)].

(ii) S_Q_from_model receives a model as a dol file and computes the theoretical structure factor associated with the model after orientation averaging [equation (5)]. To S_Q_from_model, we added an option to include the effect of a finite temperature by averaging over *N*_*T*_ different random configurations (subunit positions, **r**_*i*_, using thermalize), assuming thermal fluctuations, 

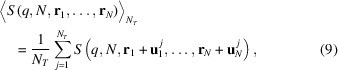

where 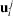 is the *j*th displacement in the position **r**_*i*_ of the *i*th subunit. This function can better model realistic samples with thermal fluctuations. The displacements assume a Gaussian distribution of average standard deviation 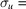 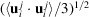 .

(iii) S_Q_from_I computes the structure factor from the number of subunits, *N*, the total intensity, *I*, and the form factor of a subunit. The structure factor of oriented samples is a double sum over the complex exponents of the projections of the distances between all subunit pairs on the scattering vector (*i.e.* the phase factors) without the form-factor coefficients. The scattered intensity from an oriented sample containing copies of the same subunit is 

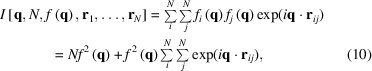

where the *i*th subunit form factor *f*_*i*_(**q**) is equal to *f*(**q**) and **r**_*ij*_ = **r**_*i*_ − **r**_*j*_, and the structure factor is given by equation (4). In isotropic solutions, we average over all the orientations and get a 1D structure factor [equation (5)] or

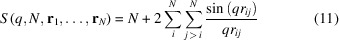

that is implemented in *D*+ because it is somewhat faster to compute.

Using the second function in equation (10), we can approximate the structure factor from a solution scattering curve, *I*, of a system with *N* identical subunits, provided we have the solution scattering curve of the subunit (form factor) |*f* ^2^(*q*)|: 

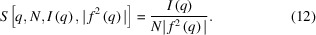


**Figure 1:**
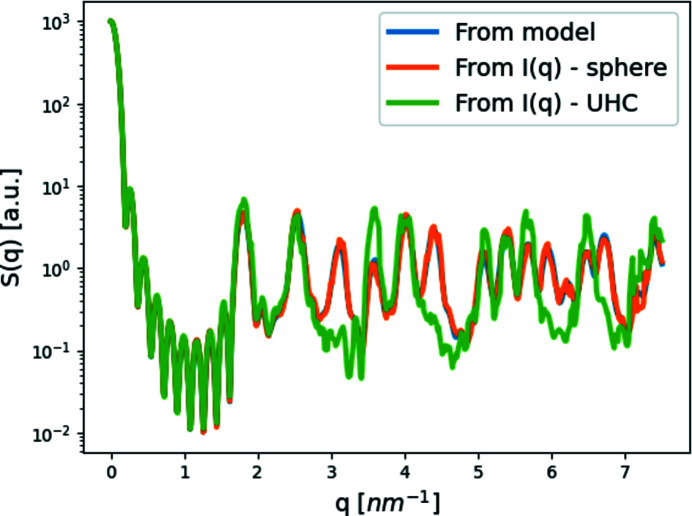
Examining the assumption behind structure factor, *S*(*q*), computations. *S*(*q*) of our cubic crystal model (lattice parameter *a* = 3.5 nm, and ten subunit repetitions in each axis) was computed in three ways. We first computed *S*(*q*) from a list of subunit point locations [equation (11), blue curve]. The other two structure-factor curves were obtained using equation (12). Initially, the scattered intensity of the same crystal was computed, where the subunits were either symmetric (spheres with a radius of 1.5 nm) or asymmetric (cylinders with a height of 3.2 nm and a radius of 0.17 nm). We then divided each scattering intensity by its subunit form factor: sphere (orange curve) or cylinder (green curve). Except for some discrepancies owing to numerical errors, the difference between the crystal model (blue curve) and the symmetric subunit (sphere – orange curve) graph is minimal, unlike the difference between the crystal model (blue) and the asymmetric subunit (cylinder – green) graphs. The results demonstrate the assumption behind equation (12) stating that the form factor has to come from subunits with spherical symmetry.

S_Q_from_I receives the number of subunits, *N*, the intensity and the subunit form factor (rather than a list of subunit positions), and computes equation (12). Therefore, the intensity must be correctly normalized to the absolute intensity and the density of subunits. Equation (12) assumes that the subunits are spherically symmetric. If not, deviations from the exact structure factor [equation (11)] appear (Fig. 1 ▸).

(iv) s_q_from_g_r converts a given radial distribution function, *g*(*r*), into a structure factor using the known relation between the two (Als-Nielsen & McMorrow, 2011 ▸; Hansen & McDonald, 2013 ▸; Egelstaff, 1992 ▸): 

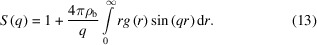

This function can be implemented using either *SciPy*’s discrete sine transform (DST) (Virtanen *et al.*, 2020 ▸) or the numerical Simpson integration method. Equation (13) has integration to infinity but the cut-off is determined by the *r*_max_ value of the radial distribution function.

In addition, using the Python API of *D*+, it is possible to compute any other structure-factor function (Baxter, 1970 ▸), fill an amplitude grid (using f ill), and multiply it by the amplitude grid of the form factor (Amp_multi). The resulting amplitude can then be loaded to *D*+ or a state file and ‘Generate’ can be used to square the amplitude and compute its orientation average.

For polydispersed subunits, it is possible to implement equation (3) using the GUI of *D*+ or the Python API of *D*+. Equation (4) can be computed at each reciprocal grid amplitude point using the Python API of *D*+. The structure-factor amplitude grid can then be multiplied (Amp_multi) by any form-factor amplitude grid and added (Amp_sum) to other amplitude grids, representing other structures. The last two structure-factor options are demonstrated in a Jupyter Notebook (https://github.com/uri-raviv-lab/dplus-dev/blob/development/PythonInterface/getting_started.ipynb).

#### 2.4.3. *g*(*r*)

The radial distribution function is another tool often used to analyze X-ray data to study how local densities vary in a crystal or a liquid. From these data, one can understand the general structure and symmetries in a sample (Olgenblum *et al.*, 2020 ▸; Mu *et al.*, 2019 ▸; Yarnell *et al.*, 1973 ▸). Our module contains two functions:

(*a*) g_r_from_s_q computes the radial distribution function from a structure factor by reordering equation (13) and applying the inverse Fourier transform (Als-Nielsen & McMorrow, 2011 ▸; Biehl, 2019 ▸; Hansen & McDonald, 2013 ▸): 

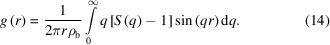

This function encounters the same problems as its twin [equation (13)] and is computed up to a cut-off determined by the value of *q*_max_.

(*b*) g_r_from_model receives a model (a list of subunit positions, *i.e.* a dol file) and finds the radial distribution function through a simple binning technique around a randomly chosen point of reference [equation (6)]. This function can either count the number of subunits between *r* and *r* + Δ*r* (assuming the subunits are points) or take into account the subunit size (or radius) and compute the exact total volume of subunits between *r* and *r* + Δ*r*. It is possible to average different configurations (using one of the supporting functions) and/or different initial subunits of reference within the same configuration.

### 2.5. 2D scattering intensities

The 2D scattering intensity from oriented structures or fibers can be computed using the Python API of *D*+. First, one must calculate the 3D reciprocal grid scattering amplitude (using the GUI of *D*+ or its Python API). One can then use the Python API to calculate 2D scattering patterns from fibers or oriented structures (as in crystallography experiments). This can be done by positioning the structure in a specific orientation with respect to the *y* axis (which is the beam direction).

The user must also define the 2D pattern density by providing the total number of calculated points along each detector axis (from the negative to the positive side). This number is used for both the *q*_⊥_ and *q*_*z*_ axes. In other words, the size of the returned 2D matrix will be the number of calculated points squared. The calculation time can thus quickly become very large. Interpolations are used to compute the amplitudes at points in the 2D matrix between precomputed reciprocal grid points.

In the current version of *D*+, to calculate 2D intensities one needs to use a reciprocal grid from the leaves up to the root of the model tree data structure. This means that the advantages of the hybrid method cannot be used yet, and large grids and reasonable computation power might be needed for computing the 2D scattering pattern from large structural models. This limitation will be removed in a future version of *D*+.

#### 2.5.1. Single orientation

A function (get_crystal_intensity) has been written to simulate the 2D scattering intensity from a structure in a single orientation (as in crystallography experiments), whose input is a reciprocal grid amplitude computed by *D*+. Amplitudes are kept as a grid with polar coordinates in reciprocal space, *F*(*q*, θ_*q*_, ϕ_*q*_). In experiments with oriented samples, the 2D detector intersects with the sample’s Ewald sphere (represented by the amplitudes) at a specific ϕ_*q*_. The default ϕ_*q*_ value is 0 for positive *q*_⊥_ values and π for negative *q*_⊥_ values. Other ϕ_*q*_ values can be calculated if needed, in which case, for negative *q*_⊥_ values, ϕ_*q*_ + π is used instead of ϕ_*q*_. The function gets the number of calculated points and generates the 2D matrix in (*q*_⊥_, *q*_*z*_) space, and each pixel is converted to polar coordinates: 

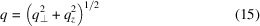

and

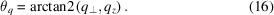

The 2D scattering intensity, |*F*(*q*, θ_*q*_, 0)|^2^ for positive *q*_⊥_ and |*F*(*q*, θ_*q*_, π)|^2^ for negative *q*_⊥_, is then obtained by interpolations from the 3D reciprocal grid of the root.

As the 3D reciprocal grid amplitude contains all the ϕ_*q*_ values, it is possible, using the same amplitude, to sample other planes of the structural model around the *z* axis by inputting a different ϕ_*q*_ value, 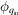 , for positive *q*_⊥_. In such a case, amplitudes at negative *q*_⊥_ values are calculated at 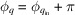 .

#### 2.5.2. Fiber diffraction

In fiber diffraction experiments, the scatterers are aligned in the polar angle in real space, θ_r_, and isotropically distributed around the azimuthal angle in real space, ϕ_r_. The following azimuthal average, therefore, gives the scattering intensity in reciprocal space: 

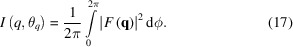

 To compute this average, the API function get_fiber_intensity uses *D*+’s Monte Carlo integration function in which equation (16) becomes

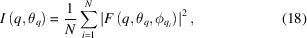

and 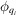 is randomly and uniformly sampled in the distribution [0, 2π) by the use of the relationship ϕ = 2π*u* and *u* ∈ [0, 1). The number of sampled ϕ_*q*_ values grows until convergence is attained according to the Monte Carlo algorithm of *D*+ (Ginsburg *et al.*, 2019 ▸). We also added the option to change the sampling domain to a user-defined one, simulating a sample with only certain azimuthal orientations. The sampling equation then becomes 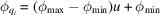 .

## 3. Usage examples

Several usage example codes and graphs can be found in our Jupyter Notebook. Some of the examples are discussed in this section.

### 3.1. Supporting functions

With these functions, we first built a simple cubic crystal with a lattice parameter *a* = 3.5 nm and ten repetitions in each axis (*i.e.* a 10 × 10 × 10 lattice), which will be the model used in our examples.

**Figure 2:**
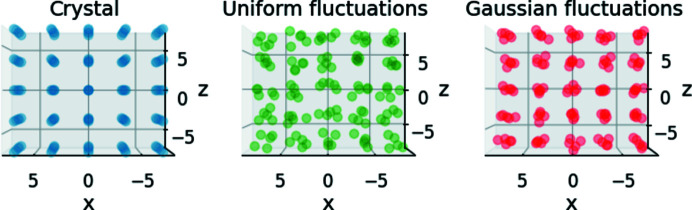
Our cubic crystal. (Left panel) A view of a 5 × 5 × 5 section of our simple cubic crystal model with a lattice parameter *a* = 3.5 nm and 10 × 10 × 10 subunits. This is the model used in our examples. (Center panel) A view of the same model, but, to each lattice point, a random fluctuation was added in all directions according to a uniform distribution within the domain [−0.3 nm, 0.3 nm). (Right panel) A view of a simple cubic crystal to which random fluctuations were added in all directions according to a Gaussian distribution with a mean at the lattice point coordinate and a standard deviation, σ_*u*_, of 0.3 nm.

We then added thermal fluctuations to the same cubic crystal. We added displacements that are either uniformly distributed in the domain [−0.3 nm, 0.3 nm) [where the half-opened domain stems from the way random numbers are generated, as explained by Press *et al.* (2007 ▸)] or according to a Gaussian distribution with a standard deviation, σ_*u*_, of 0.3 nm. In both cases, fluctuations were applied to all three axes. Fig. 2 ▸ shows a partial side view of the resulting crystals (shown as a 5 × 5 × 5 cube for clarity).

### 3.2. Resolution function

**Figure 3:**
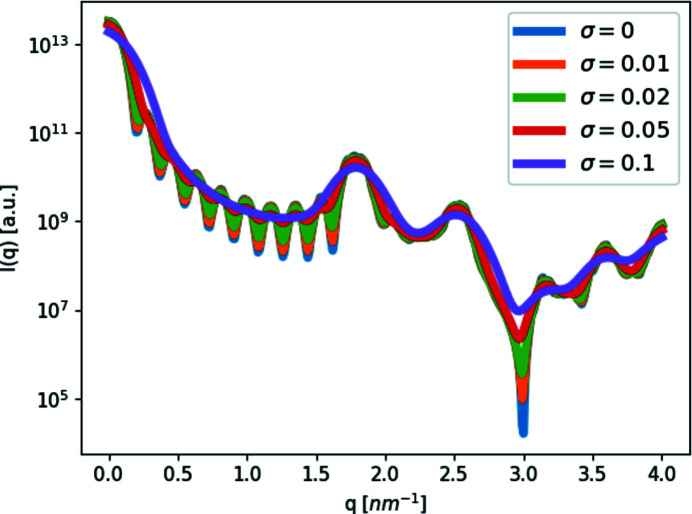
Effect of the instrument-resolution function. Scattering intensity, *I*, as a function of the magnitude of the scattering vector, *q*, from our simple cubic crystal (*a* = 3.5 nm and 10 × 10 × 10 subunits), with spheres of radius 1.5 nm as subunits, to which a Gaussian resolution function was added with four different standard deviation values: σ_r_ = 0.01, 0.02, 0.05 and 0.1 nm^−1^. As σ_r_ grows, the peaks start to merge and the extrema become less localized.

To demonstrate how the resolution function, computed as explained (Ben-Nun *et al.*, 2010 ▸), changes the scattering curve, we computed the scattering curves from spheres with a radius of 1.5 nm, arranged in our cubic crystal model. We calculated the scattering curve in *D*+, with and without a resolution function. We used typical σ_r_ values of 0.01 and 0.02 nm^−1^ but also some extreme values of 0.05 and 0.1 nm^−1^ that better demonstrate the effect. As expected, the higher the σ_r_, the less localized and prominent the peaks and minima are (Fig. 3 ▸).

### 3.3. Polydispersity

**Figure 4:**
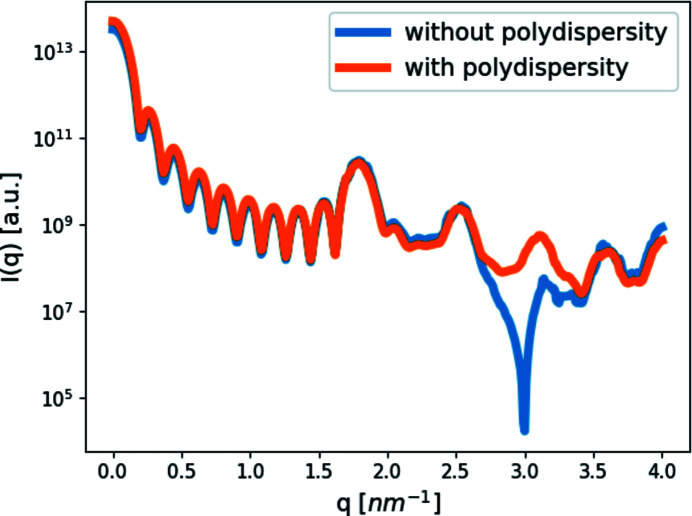
Effect of polydispersity. Scattering curves of our simple cubic crystal (*a* = 3.5 nm and 10 × 10 × 10 subunits), with spheres of radius 1.5 nm as subunits. We then added a Gaussian distribution function with a standard deviation of 0.3 nm to the radius of the spheres (with a mean at the inputted radius) to simulate the effect of polydispersity in the model. The minima, related to sphere radius, have shifted and are a lot less prominent, as would be expected from a polydispersed system.

Similarly, we ran the same model (without a resolution function) and added a Gaussian polydispersity with σ_p_ of 0.3 nm around the mean 1.5 nm sphere radius. In other words, *D*+ computed multiple times the scattering intensity from spheres with radii selected from a Gaussian weighting distribution with a mean of 1.5 nm and a standard deviation of 0.3 nm (Fig. 4 ▸). In addition to filling the sharp minima as in Fig. 3 ▸, the positions of the minima and maxima have shifted slightly.

### 3.4. *S*(*q*) and *g*(*r*)

This section demonstrates different ways to compute the structure factor and how they affect the result. In addition, we examined the effect the spherical symmetry assumption has on the result shown in Fig. 1 ▸. For this, we used a sphere with a radius of 1.5 nm as one model, and a cylinder with a height of 3.2 nm and a radius of 0.17 nm as a second model. We show that with the cylinders, already at *q* ≃ 3 nm^−1^, the assumption becomes critical for further analysis of the model.

**Figure 5:**
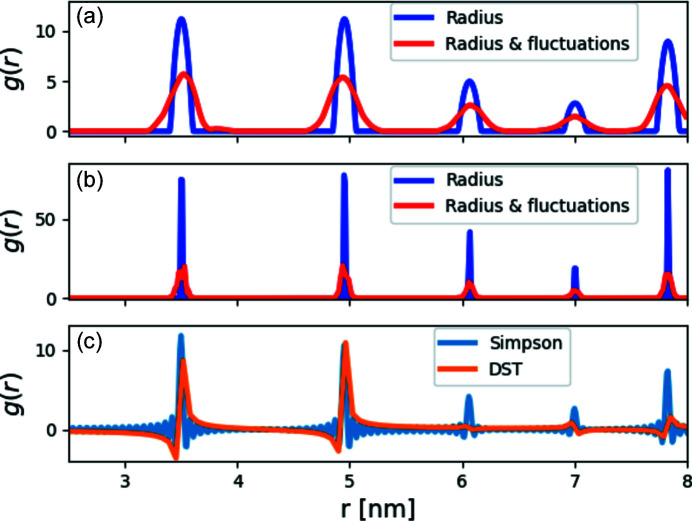
Radial distribution functions, *g*(*r*). (*a*) The radial distribution function from a 10 × 10 × 10 cubic crystal model (*a* = 3.5 nm), calculated according to equation (6) with spherical subunits with a radius of 0.1 nm, with (red curve) and without (blue curve) thermal fluctuations (σ_*u*_ = 0.1 nm). (*b*) Repeating the calculation in (*a*) with a subunit radius of 0.01 nm, with (red curve) and without (blue curve) thermal fluctuations (σ_*u*_ = 0.03 nm). We added a radius to the subunit lattice points to avoid delta functions at the peak positions and get results that are closer to reality. (*c*) The radial distribution function of the same crystal model (assuming the subunits are points), without thermal fluctuations, computed by equation (14) [using *S*(*q*) from Fig. 6 ▸ (blue curve), computed until a *q*_max_ of 100 nm^−1^], using Simpson’s integration or DST.

Fig. 5 ▸ shows the calculated radial distribution function of our crystal [equation (6)], assuming the particles have a radius of 0.3 nm (using g_r_from_model), with and without thermal fluctuations (using thermalize). We compared it with the *g*(*r*) calculated from our cubic crystal model, assuming the spheres are delta functions [Fig. 5 ▸(*b*)]. As expected, both radial distribution functions calculated from the model returned peaks at the typical distances for our cubic crystal, where the first three are at

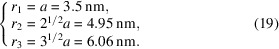

However, when computing the radial distribution function from a structure factor [equation (14)], the results [Fig. 5 ▸(*c*)] are not as good as in the blue curve of Fig. 5 ▸(*b*). We get the peaks with Simpson’s algorithm, but they are relatively wide and not always perfectly centered around the correct value. With the DST algorithm, we only get some of the peaks, whose maxima are sometimes off the expected values. It is, therefore, better to use Simpson’s algorithm. Another difference between the model radial distribution function [equation (6)] and the integrated radial distribution function from a structure factor [equation (14)] is the intensity of the peaks. However, the area under the peaks, 

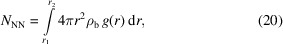

revealing the total number of nearest neighbors (nn_t_) (Als-Nielsen & McMorrow, 2011 ▸), is better preserved than the peak line-shape intensity. After integrating up from *r*_1_ = 3.4 to *r*_2_ = 3.6 nm, we found that the number of nearest neighbors (which is expected to be 6) is 5.90, 5.71 and 5.30 for the radial distribution function calculated from the model [equation (6)], Simpson’s integration and the DST, respectively. Specifically for the DST, we integrated over the range [3.5, 3.7], because otherwise we got an incorrect *N*_NN_ owing to the negative oscillation before the peak.

**Figure 6:**
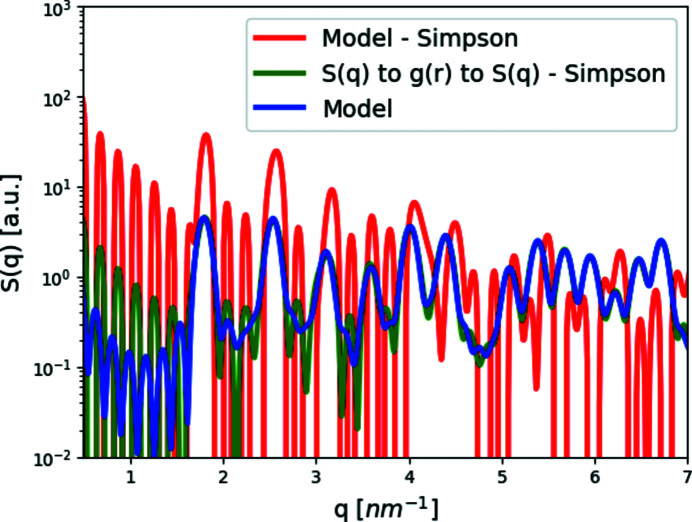
Comparing the structure factor emanating from the radial distribution function [equation (6), *r*_max_ = 32.5 nm] of a cubic crystal (*a* = 3.5 nm with 10 × 10 × 10 subunits), using equation (13) and Simpson’s integration (red curve), with the proper structure factor [equation (5), blue curve]. The green curve was obtained by taking the structure factor [equation (5), blue curve], Fourier transforming it into a radial distribution function [equation (14), using *q*_max_ = 8 nm^−1^], and then changing it back to the structure factor, using Simpson’s integration method [equation (13), using *r*_max_ = 32.5 nm].

Lastly, using equation (13), we tried to return to the structure factor [equation (5), Fig. 6 ▸, blue curve] from the radial distribution function calculated from a model [equation (6), Fig. 6 ▸, red curve] or a structure factor [equation (14), Fig. 6 ▸, green curve]. Fig. 6 ▸ shows that neither function was able to completely reproduce the modeled structure factor [equation (5), Fig. 6 ▸, blue curve]. However, when we took the structure factor [equation (5)], Fourier transformed it to the radial distribution function [equation (14)] using Simpson’s integration method, and then returned it to *S*(*q*) [equation (13), Fig. 6 ▸, green curve] using the same integration method, the deviation from the modeled structure factor [equation (5), Fig. 6 ▸, blue curve] was rather small.

*D*+ allowed us to carefully examine the assumptions often applied when computing structure factors and radial distribution functions. We also demonstrated that care must be taken when analyzing the results as they can significantly deviate from the correct values.

### 3.5. Fiber diffraction and single orientation

To demonstrate the most important upgrade of the Python API of *D*+, we used a model of graphene and computed its 2D scattering pattern. This model was used because it is a 2D structure, and thus, the direction of the beam could be determined. Another reason was that the lattice parameters are known in real space and thus also in reciprocal space, meaning the resulting diffraction can be validated. The graphene model was built to be on the *xz* plane and used the following real-space lattice vectors (Wallace, 1947 ▸; Yang *et al.*, 2018 ▸): 

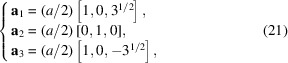

where *a* = 0.246 nm. The crystal had 25 repetitions in the **a**_1_ and **a**_3_ directions and only one repetition (to get a 2D structure) in the **a**_2_ direction (which is parallel to the direction of the beam). The lattice vectors in reciprocal space are 

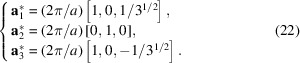

**Figure 7:**
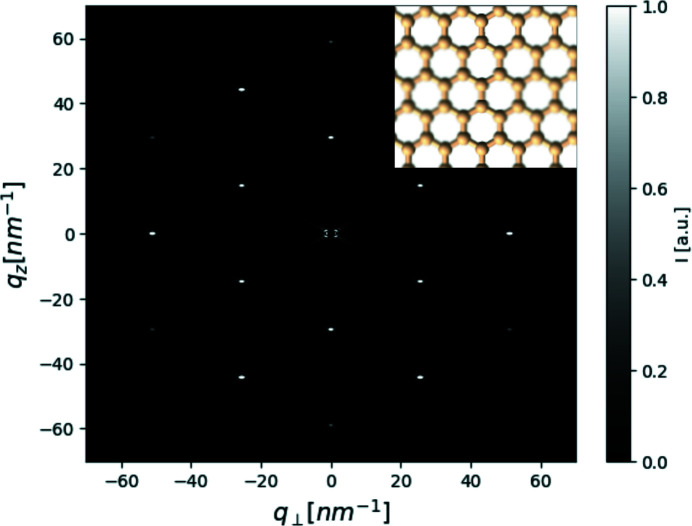
A simulated 2D scattering pattern from a layer of graphene (containing 25 × 25 unit cells; partly shown in the inset) aligned parallel to the *xz* plane (where the beam is along the *y* axis). The high intensity around *q* = 0 has been blacked out to emphasize the correlation peaks at higher *q* values. To make the different peaks more prominent, the intensities were rescaled using *scikit*’s (van der Walt *et al.*, 2014 ▸) rescale_intensity function. The simulated peaks fall on the peaks expected from the reciprocal lattice of graphene [equation (22)].

Fig. 7 ▸ shows the 2D scattering pattern, which falls on the theoretically expected Bragg peaks. This demonstrates the single-orientation scattering simulation capabilities of *D*+.

**Figure 8:**
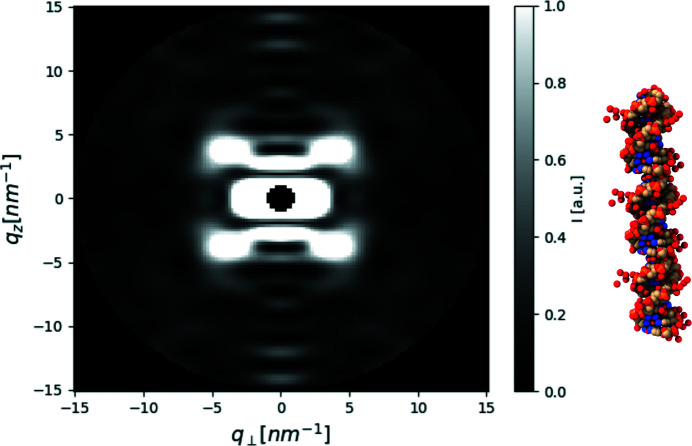
Computed fiber diffraction of a B-DNA model (using PDB ID 1zew). The typical ‘X’ 2D scattering pattern from double helices can be seen. The intensities were rescaled using the rescale_intensity function and a beamstop was added at the low *q* values.

To simulate a fiber diffraction experiment, we built a model whose base leaf is a PDB entry for DNA (Protein Data Bank; PDB ID 1zew; Hays *et al.*, 2005 ▸) inside a Manual Symmetry that added two other subunits, 3.5 nm above and 3.5 nm below the original unit. The PDB unit was aligned parallel to the *z* axis by rotating it with angles γ = 60.5° around the *z* axis and α = 90° around the *x* axis. The result of the simulation (Fig. 8 ▸) clearly shows the expected typical ‘X’ pattern of helical structures (Cochran *et al.*, 1952 ▸). The computation time of a single orientation is about three orders of magnitude faster than the computation time of fiber diffraction (though this can vary with the convergence parameters of *D*+).

## 4. Conclusions

In this work, we have reported the functions and capabilities added to *D*+, some of which are implemented in the GUI and all in the Python API of *D*+. To the GUI (and the API), we added the possibility to add a resolution function to the resulting intensity and take into account the setup resolution. To the API, we added the possibility of taking into account the polydispersity of geometrical models. In addition, we added a module for computing the structure factor and the radial distribution function from either scattering data or a structural model, including the effects of thermal fluctuations and intermolecular interactions. Lastly, we added functions that can compute the 2D scattering pattern from either a single orientation or fibers. As *D*+ can create and compute highly complicated hierarchical structural models, these additions open new opportunities for modeling complex fiber and crystallographic structures. In future updates of *D*+, most changes will be implemented in the Python API of *D*+ owing to its wide versatility.
